# Oteando la educación para la salud en el sistema de salud colombiano

**DOI:** 10.15446/rsap.V25n3.102899

**Published:** 2023-05-01

**Authors:** Nelly Johanna Loboa-Rodríguez, Diana Paola Betancurth-Loaiza

**Affiliations:** 1 NL: Enf. Esp. Gerencia de la Salud Pública. M. Sc. Salud Pública. M.Sc. Investigación de la Atención Primaria en Salud. Universidad de los Llanos, Villavicencio, Colombia. johannaloboa@unillanos.edu.co Universidad de los Llanos Investigación de la Atención Primaria en Salud Universidad de los Llanos Villavicencio Colombia johannaloboa@unillanos.edu.co; 2 DB: Enf. Esp. Promoción de la Salud. Esp. Administración en Salud. M.Sc. Enfermería. M.Sc. Investigación de la Atención Primaria en Salud. Ph. D. Salud Pública. Universidad de Caldas, Manizales, Colombia. diana.betancurth@ucaldas.edu.co Universidad de Caldas Salud Pública Universidad de Caldas Manizales Colombia diana.betancurth@ucaldas.edu.co

**Keywords:** Sistemas de salud, salud pública, política pública, educación en salud, enfoque pedagógico *(fuente: DeCS, BIREME)*, Health systems, public health, public policy, health education, pedagogical approach *(source: MeSH, NLM)*

## Abstract

Desde el establecimiento del Sistema Nacional de Salud en la década de 1970, pasando por la institucionalización del Sistema General de Seguridad Social en Salud y sus reformas, la educación para la salud en las acciones de salud pública ha estado situada en la normatividad, principalmente desde la perspectiva tradicional. La transformación de la política pública en salud, en los últimos años, a la luz de la promulgación de la salud como un derecho fundamental, ha sugerido avanzar hacia prácticas de educación para la salud instaladas en el enfoque crítico; sin embargo, no existen procedimientos específicos para la ejecución, lo cual es un reto, porque estructuralmente el sistema de salud funciona aún de acuerdo con la lógica de mercado sustentada en la teoría neoliberal utilitarista.

## Abstract (en)

From the establishment of the National Health System in the 70s of the 20th century, going through the institutionalization of the general system of social health security and its reforms, health education in public health actions has been located in the regulations, mainly from the traditional perspective. The transformation of public health policy in recent years of the current century, in light of the promulgation of health as a fundamental right, has suggested moving towards health education practices located in the critical approach; however, there are no specific procedures for implementation, and it is a challenge, because structurally the health system still works, according to the logic of the market supported by utilitarian neoliberal theory.

La educación para la salud es una práctica social que, en el contexto de lo pedagógico, ha sido abordada desde concepciones no homogéneas 1, y en ello sobresale la tensión entre los modelos tradicional y crítico. El primero, relacionado con una concepción positivista de la ciencia, de tipo biomédico de la salud, y, por su parte, el modelo crítico, sustentado en una comprensión de la ciencia y la salud como producto de las condiciones sociales, culturales y ecológicas.

En el contexto del sistema de salud colombiano, la educación para la salud ha estado incluida en diferentes políticas públicas, y ha sido propuesta tanto desde la perspectiva tradicional como desde la crítica. A continuación, se presenta un ensayo que explora esta práctica social, en el escenario normativo de la salud pública, en el interior del sistema de salud, en una cronología que abarca el último tercio del siglo XX hasta la actualidad.

Es este un aporte a la reflexión que, como sostiene Ospina 2, es importante realizar con respecto a la normatividad de la acción política por la salud, para comprender las decisiones estatales y suscitar análisis en relación con los marcos legales que regulan la sociedad a partir del sistema de salud.

## Pistas sobre educación para la salud en acciones de salud pública del Sistema Nacional de Salud

La legislación que ha emergido para desarrollar las acciones de salud pública en Colombia, desde el último tercio del siglo pasado hasta la actualidad, ha evolucionado de acuerdo con los planes de gobierno presidenciales, en consonancia con las directrices de orden internacional asociadas al modelo de Estado legitimado.

Antes de 1975, las demandas sanitarias de tipo poblacional en el país eran atendidas por un conjunto desarticulado y heterogéneo de instituciones, cuyos servicios estaban aglomerados, principalmente, en programas de erradicación y control de enfermedades transmitidas por vectores, así como de prevención de enfermedades inmunoprevenibles, que contaban con la participación activa de las comunidades y algunos apoyos financieros de tipo internacional.

El Sistema Nacional de Salud (en adelante SNS) se estableció en la década de 1970, con la promulgación del Decreto 056 3, por medio del cual se reflejó una concepción sistémica inspirada en los Estados europeos del bienestar. Se concretó así, por primera vez en la nación, una estructura institucional relacionada con un modelo de atención, en el que la salud pública se comenzó a comprender como un sector estatal encargado de la salud de toda la población y no solamente de la atención y la prevención de epidemias 4.

En el SNS surgieron los servicios seccionales de salud de los diferentes territorios departamentales, que trabajaban como dependencias técnicas del Ministerio de Salud Pública, cumpliendo con funciones como adaptar y adoptar la política nacional de salud para atender las características tan disímiles de cada región.

Para descentralizar la planeación y ejecución de acciones en el sector salud, en 1990 se emitió la pauta normativa que otorgó autonomía a los departamentos y municipios 5; a partir de esto, se reorganizó el SNS, para asignar como función principal a las direcciones locales de salud, la formulación de los planes de salud con asignación de recursos. De tal manera, se inició un proceso de planeación local participativa en la salud, establecida con la Constitución Política de 1991 6.

El SNS abarcaba tres subsectores separados e independientes: el público, el de seguridad social para los trabajadores formales y el sector privado. Glassman *et al.*^7^ describen que el financiamiento del subsector público estaba orientado a los hospitales públicos; la atención primaria; los programas verticales destinados a combatir el paludismo, la tuberculosis y la lepra; los planes de vacunación y la salud materno-infantil y reproductiva, así como el control de enfermedades. Además de los programas verticales, no existía ninguna asignación separada de recursos para la prevención de enfermedades y actividades de promoción de la salud o actividades de salud comunitaria.

Así, las acciones de salud pública en el SNS se relacionaron, principalmente, con programas de atención dirigidos al control y la prevención de enfermedades transmisibles. La educación para la salud se desarrollaba a partir de un enfoque tradicional, a manera de estrategia para transmitir a la comunidad conocimientos sobre el cuidado de la salud, como también sobre la identificación de signos y síntomas de enfermedades, con logros significativos, pero subvalorados cuando se estableció el Sistema General de Seguridad Social en Salud, y que fueron suspendidos, con repercusiones negativas en los indicadores de salud pública.

Un ejemplo de esto último lo constituyó el programa de control de tuberculosis, que durante el SNS fue dirigido verticalmente por el Ministerio, con el tamizaje obligatorio de los sintomáticos respiratorios con bacilos-copias seriadas, captación de enfermos, suministro de esquemas terapéuticos supervisados, vacunación de todo recién nacido con la BCG y educación para la salud desde un enfoque preventivo. El riesgo de infección por tuberculosis disminuyó en la década de 1970, y se mantuvo bajo hasta los años noventa, cuando se observó un incremento en la incidencia 8.

Con el SNS se fortaleció y favoreció tanto la capacitación, como la contratación a escala nacional de mujeres que eran educadoras sanitarias en sus propias veredas. Esto, con el Programa de Promotoras Rurales de salud, que surgió años antes, por iniciativa del profesor Héctor Abad Gómez 9, y formó a actores clave para la implementación de la estrategia de Atención Primaria en Salud (APS) en las zonas rurales y apartadas del país 10.

Con la reglamentación del ejercicio de la enfermería en el país, las promotoras de salud fueron incorporadas como la base del SNS 11 y constituyeron una parte fundamental de los centros de salud que más tarde conformaron las unidades primarias de atención (UPA). Así, se dinamizó la implementación de diferentes aspectos de la Atención Primaria en Salud (APS), sugeridos en Alma Ata 12.

## La educación para la salud en las acciones de salud pública del Sistema General de Seguridad Social en Salud

Restrepo 13 afirma que una de las reformas más profunda y ambiciosa que se ha aplicado sobre un sector social en Colombia ha sido La ley 100 14, con la que se organizaron los servicios de salud, desde criterios de mercado, y se creó el Sistema General de Seguridad Social en Salud (SGSSS). Así, el rol que se asignó al Estado fue el de corregir las fallas del mercado.

El SNS se transformó en un SGSSS, con el propósito de generar cobertura global mediante un sistema de aseguramiento nacional y obligatorio, buscando el aumento de eficiencia.

Las acciones de este sistema se fundamentaron en el modelo de desarrollo neoliberal, coherente con el modo de producción capitalista, lo que para el sector salud configuró una visión de justicia de tipo individualista, ligada a la propiedad, la distribución individual y la focalización de recursos públicos 15, con una figura de Estado protector del mercado y la libre competencia para promover el avance económico como condición para el desarrollo social y humano 16.

En la Ley 100 de 1993 14, el término salud pública no se enunció en ninguno de sus apartados constitutivos; en cambio, se especificaron intervenciones dirigidas a los colectivos poblacionales; así, en el capítulo III se definió el Plan de Atención Básica (PAB) como el conjunto de actividades dirigidas a toda la población de manera gratuita, sin estar sujeto a ningún tipo de afiliación en salud. Las acciones de salud pública se surtieron como complemento a las acciones del Plan Obligatorio de Salud (POS).

El PAB se formalizó mediante la Resolución 4288 de 1996 17, y con su puesta en marcha se generó un impacto en el manejo de la salud pública desde el direccionamiento gubernamental, al dar apertura a la articulación de las acciones de sectores diferentes al de salud y fuerzas vivas de la sociedad, para la ejecución de intervenciones que permitieron la implementación de las cinco macroestrategias de la carta de Ottawa.

La gestión del PAB entre los años 2001 y 2007 desarrolló un modelo por procesos ligados a la planeación territorial, con prioridades, acciones, metas e indicadores nacionales en salud pública, de obligatorio cumplimiento por parte de los entes gubernamentales.

Por medio del PAB se daba respuesta a indicadores de proceso, relacionados con número de actividades realizadas y de personas convocadas. Así, la práctica de la educación para la salud se desarrolló principalmente de modo tradicional, mediante prácticas transmisionistas de información, principalmente unidireccionales y de corto plazo, enfocadas en el cambio de comportamientos, sustentada en modelos pedagógicos convencionales desarrollados con facilidad, dentro de la lógica del modelo hegemónico biomédico.

A partir de lo anterior, los indicadores requeridos para evidenciar la gestión del PAB no serían los relacionados con la disminución de la morbimortalidad, sino los que mostraran la cantidad de actividades realizadas, en contraste con las proyectadas, con una lógica mercantilista en la cual el rendimiento del talento humano se midió por la eficacia de su quehacer y no por el impacto de la implementación de sus competencias como aporte al mejoramiento de la calidad de vida y la salud de las personas, las familias y las comunidades.

En consecuencia, los procesos no exigieron una medición de sus efectos sobre las comunidades, que permitiera dimensionar el alcance de la promoción de la salud mediante la aplicación de la educación para la salud, sino el cumplimiento concreto de actividades. Así, los servicios de salud, categorizados como bienes de transacción, implicaron una educación para la salud instrumentalizada, a favor del reporte de indicadores de cumplimiento.

## La educación para la salud según las reformas al Sistema General de Seguridad Social en Salud

En el 2007 se introdujeron modificaciones al SGSSS, al establecerse su primera reforma con la Ley 1122 18, de la cual surgió el Plan Nacional de Salud Pública (PNSP) para períodos de 4 años. Se definió a la salud pública como un conjunto de políticas que, bajo la rectoría del Estado, buscan garantizar, de una manera integrada, la salud de la población por medio de acciones de salubridad dirigidas de manera individual y colectiva.

En este contexto, la educación para la salud se ha delimitado como una herramienta de promoción de la salud, caracterizada como un instrumento de gestión pública territorial para el manejo de recursos humanos, logísticos y financieros, mediante la práctica pedagógica tradicional dirigida a poblaciones, vulnerables o en riesgo, de acuerdo con las tendencias de los perfiles epidemiológicos, los análisis de situación en salud de cada territorio y la agenda internacional.

En la Ley 1122 18 se estipuló que los planes debían tener una vigencia de cuatro años, sin embargo, con su primera ejecución, se concluyó que un cuatrienio era poco tiempo para diseñar y aplicar políticas que evidenciaran resultados de mediano y largo plazo.

En consecuencia, se reformó nuevamente el SGSSS, al emitirse las directrices para formular el Plan Decenal de Salud Pública, por medio de la Ley 1438 del 2011 19, y en el 2013 se expidió el Plan Decenal de Salud Pública (PDSP 2012-2021) 20.

Para materializar a escala territorial el PDSP 2012-2021, en el 2015 se establecieron los lineamientos del Plan de Salud Pública de Intervenciones Colectivas (PSPIC) 21, como parte integral del Plan Territorial de Salud (PST). Lo anterior, con el propósito de impactar positivamente en los determinantes sociales de la salud (DSS), con la ejecución de intervenciones dirigidas a poblaciones a lo largo del curso de la vida, principalmente por medio de la educación para la salud.

En el año 2016, entre las directrices para la formulación de los PSPIC, se definió una intervención denominada educación y comunicación para la salud 22. Con esta, el Ministerio de Salud y Protección Social generó una guía con orientaciones políticas y teóricas, en la que se describía la educación para la salud de conformidad con los conceptos emitidos en Ottawa, es decir, como una forma de hacer las cosas desde la promoción de la salud, sin fundamentarse únicamente en el modelo pedagógico tradicional. Se sugirió una educación para la salud orientada hacia acciones participativas, con abordaje de ámbitos específicos, a fin de proponer alternativas de cambio con las comunidades.

Lo anterior, a partir de los postulados del modelo educativo interestructurante-dialogante, con el propósito de dirigir la acción pedagógica hacia la transformación de la realidad, para conducir a mejores niveles de práctica, empleando instrumentos cognitivos, valorativos y prácticos, desde el carácter histórico, social y cultural de los individuos y sus saberes; todo esto, sustentado en el modelo pedagógico crítico.

Sin embargo, este enfoque excede la esencia del SGSSS, situado en la hegemonía biomédica y el enfoque mercantilista, que concibe a las personas como usuarios, pacientes o receptores de intervenciones, y las reconoce como destinatarias de intervenciones y no como sujetos con quienes establecer diálogos de saberes. Esto último, no obstante, es necesario para actuar desde una postura crítica de la educación para la salud.

Es escasa la evidencia que visibiliza el desarrollo de prácticas de educación para la salud en las bases de la perspectiva crítica. Es posible que su desarrollo sea reducido, al estimular la generación de procesos emancipatorios contra el mismo sistema del que surge y, además, porque se sitúa en planes de salud pública que privilegian la focalización de grupos poblacionales, sin reconocer las expectativas, las necesidades sentidas e incluso las realidades sociales críticas, al prevalecer el cumplimiento de las directrices protectoras de los intereses del capital.

Posteriormente, con la Resolución ministerial 429 de 2016 23, se estableció la Política de Atención Integral en Salud (PAIS), para orientar los objetivos del sistema de salud y de la seguridad social en salud, en relación con la garantía del derecho a la salud de la población. Esta legislación centró al SGSSS en los ciudadanos y no en el mercado. Se formuló gracias a la promulgación de la Ley 1751 del 2015 24, por la que se hizo más visible la necesidad de realizar cambios dentro del sistema, con el propósito de garantizar los derechos irrenunciables de la persona y la comunidad para obtener la calidad de vida, de acuerdo con la dignidad humana, mediante la protección de las contingencias que la afecten.

La PAIS 23 se estableció con un marco estratégico y uno operacional. El primero se instaló en cuatro líneas de acción, entre las que se encuentra la APS con enfoque de salud familiar y comunitaria. El marco operacional, se fundamentó en el Modelo Integral de Atención en Salud (MIAS) 25, sobre el cual Pinzón 26 expresa que fue un reconocimiento a los logros y las experiencias exitosas alcanzadas por el SGSSS, al permitir una mixtura para alinear y motivar a los actores del sistema hacia un trabajo en conjunto, en función de los resultados en salud de las personas, las familias y las comunidades como sujetos del derecho a la salud.

El MIAS 25 incorporó e integró conceptos y estrategias de probada efectividad y otras novedosas. Sin embargo, se presentaron muchas dudas con respecto a su implementación y efectividad, pues en su concepción más profunda y filosófica se enfrentó la esencia del SGSSS, relacionada con la teoría neoliberal utilitarista, en la cual la salud tiene una consideración de derecho, contrariamente a lo reafirmado por la Corte Constitucional en la Ley Estatutaria 1751 del 2015 24, en torno a la salud como derecho fundamental.

Posteriormente, con la Resolución 2626 del 2019, por la cual se modificó la PAÍS y se adoptó el Modelo de Acción Integral Territorial (MAITE) 27, se redefinió el modelo integral de atención en salud en términos de calidad, al desarrollar para tales efectos lineamientos para la implementación de modelos de atención en salud con enfoque de atención primaria en salud, salud familiar y comunitaria, territorial, poblacional y diferencial, cuya definición se delegó a cada departamento, municipio y distrito, con el propósito de orientar de forma articulada la gestión del sistema de salud en los territorios, a fin de responder a las prioridades de salud de la población y contribuir a su mejoramiento.

Para el año 2023, el Gobierno Nacional anunció una nueva reforma al sistema de salud. En caso que se apruebe y determine poner en práctica procesos de educación para la salud alternativa, se hace necesaria una transformación estructural, la cual, como afirma Peñaranda 28, gire en torno al bienestar y la salud de las personas, y no a la rentabilidad económica. Lo anterior, por medio de una práctica pedagógica que aporte de manera significativa a la comprensión de las estructuras que fortalecen o perturban la salud, originada por la interacción entre educadores y educandos 29.

Adicionalmente, se hacen necesarios cambios en pro de la cualificación del talento humano del área de la salud y las ciencias sociales, con ajustes curriculares en las universidades que, como proponen Ruiz *et al.*^30^, que fomenten el trabajo interdisciplinario e intersectorial de acuerdo con el contexto social, económico y político, para permitir a los futuros profesionales apropiarse del principio en el que la salud es resultado personal, pero, y sobre todo, social.

El contexto del sistema de salud colombiano, desde la última parte del siglo pasado, ha transitado de un modelo de Estado con rol de distribuidor igualitario de bienes sociales a un Estado de corte neoliberal.

Las acciones de salud pública, al ser intangibles y no agotarse en el consumo, por ser bienes públicos generadores de externalidades positivas, suscitan escaso interés para el mercado.

En estos términos, la legislación de política pública en salud pública se ha enfocado en la organización sistemática de planeación y ejecución de actividades. Los indicadores relevantes se centran en la ejecución de actividades y no en el establecimiento y la puesta en marcha de procesos que se reflejen en rentabilidad social, acentuándose así el desarrollo de prácticas de educación para la salud de enfoque tradicional.

En el último lustro, desde el Ministerio de Salud y Protección Social se han analizado y esbozado lineamientos que motivan la práctica de educación crítica, en el marco de la formalización de la salud como derecho fundamental, lo cual es contradictorio cuando el sistema de salud, en general, se "enraíza" en el mercantilismo de la teoría neoliberal utilitarista, y si bien el ámbito de las acciones de salud pública se ha reformado, ha sido para delimitar con mayor detalle la operatividad del rendimiento de las acciones de talento humano, y no tanto para el establecimiento de estrategias que privilegien la valoración del impacto de las intervenciones en la vida y la salud de las personas.

Si en futuras reformas al sistema de salud colombiano prevalece la consideración de la salud como un derecho fundamental, se hace necesario que la educación para la salud, además de tener lineamientos para su desarrollo, sea dimensionada como un proceso pedagógico, antes que como un instrumento de intervención ♠
